# Data‐driven motion‐corrected brain MRI incorporating pose‐dependent B_0_ fields

**DOI:** 10.1002/mrm.29255

**Published:** 2022-05-08

**Authors:** Yannick Brackenier, Lucilio Cordero‐Grande, Raphael Tomi‐Tricot, Thomas Wilkinson, Philippa Bridgen, Anthony Price, Shaihan J. Malik, Enrico De Vita, Joseph V. Hajnal

**Affiliations:** ^1^ Department of Biomedical Engineering, School of Biomedical Engineering and Imaging Sciences King's College London, St. Thomas' Hospital London United Kingdom; ^2^ Centre for the Developing Brain, School of Biomedical Engineering and Imaging Sciences King's College London, St. Thomas' Hospital London United Kingdom; ^3^ Biomedical Image Technologies, ETSI Telecomunicación Universidad Politécnica de Madrid and CIBER‐BNN Madrid Spain; ^4^ MR Research Collaborations Siemens Healthcare Limited Frimley United Kingdom

**Keywords:** motion correction, reconstruction, parallel imaging, susceptibility‐induced B_0_ variation, ultrahigh field

## Abstract

**Purpose:**

To develop a fully data‐driven retrospective intrascan motion‐correction framework for volumetric brain MRI at ultrahigh field (7 Tesla) that includes modeling of pose‐dependent changes in polarizing magnetic (B_0_) fields.

**Theory and Methods:**

Tissue susceptibility induces spatially varying B_0_ distributions in the head, which change with pose. A physics‐inspired B_0_ model has been deployed to model the B_0_ variations in the head and was validated in vivo. This model is integrated into a forward parallel imaging model for imaging in the presence of motion. Our proposal minimizes the number of added parameters, enabling the developed framework to estimate dynamic B_0_ variations from appropriately acquired data without requiring navigators. The effect on data‐driven motion correction is validated in simulations and in vivo.

**Results:**

The applicability of the physics‐inspired B_0_ model was confirmed in vivo. Simulations show the need to include the pose‐dependent B_0_ fields in the reconstruction to improve motion‐correction performance and the feasibility of estimating B_0_ evolution from the acquired data. The proposed motion and B_0_ correction showed improved image quality for strongly corrupted data at 7 Tesla in simulations and in vivo.

**Conclusion:**

We have developed a motion‐correction framework that accounts for and estimates pose‐dependent B_0_ fields. The method improves current state‐of‐the‐art data‐driven motion‐correction techniques when B_0_ dependencies cannot be neglected. The use of a compact physics‐inspired B_0_ model together with leveraging the parallel imaging encoding redundancy and previously proposed optimized sampling patterns enables a purely data‐driven approach.

## 1 INTRODUCTION

MRI is susceptible to motion that causes artifacts in reconstructed images.^1^ Prospective and retrospective motion‐correcting schemes have been proposed that enable motion‐tolerant imaging. Prospective techniques have shown to be advantageous in terms of spin history and k‐space sampling density, but the need for extra hardware limits these methods in clinical workflow.^3^ Retrospective methods, on the other hand, can be applied to a variety of sequences and do not require any type of additional hardware. The aligned sensitivity encoding (SENSE) framework^4^ achieves intrascan motion estimation and correction for volumetric anatomical imaging by temporally subdividing k‐space samples and accounting for different motion states in each segment. With an optimized Cartesian sampling pattern, later developed in the distributed and incoherent sample orders for reconstruction deblurring using encoding redundancy (DISORDER) framework,^5^ the number of segments to be considered can be increased, allowing for high temporal resolution motion correction. This method provides retrospective data‐driven motion correction for small motion levels^6^ or clinical field strengths. However, deteriorated performance was observed at increased motion levels at 7 Tesla (T), which has been attributed to susceptibility‐induced polarizing magnetic (B_0_) fields that vary with pose.^7^ When changes in B_0_ are not included in the original motion‐informed signal model,^4^ they can hamper motion‐correction techniques.

Tissue susceptibility causes the B_0_ in the head to be spatially varying, altering the signal acquired in MRI.^8^ Susceptibility‐induced B_0_ variabilities scale with field strength (approximately ±200Hz at 7 T compared to ±85Hz at 3 T within the brain) and therefore emerge as an important effect at higher field strengths. Additionally, these B_0_ fields are pose‐dependent^9^ and mainly determined by head rotations for brain MR.^10^ Various methods have been proposed to estimate these field perturbations. First, motion‐induced B_0_ variations can be predicted by forward modeling the effect of a transformed susceptibility model.^9^ This method is limited by the accuracy of the susceptibility model and ignores any other source of B_0_ variations. Second, navigators have been used extensively to measure fields before acquiring the data of interest.^11^, ^12^, ^13^, ^14^, ^15^, ^16^ A variety of 1D, 2D, and 3D navigators exists, but each of them is limited by the temporal and/or spatial resolution to provide accurate B_0_ estimates. Furthermore, scanning efficiency is often impacted by the repetitive use of navigators. Lastly, B_0_ field maps can be jointly estimated with the image from motion‐free k‐space data.^17^, ^18^ This method can be extended to motion‐corrupted data considering each segment of k‐space as a highly undersampled motion‐free image in a different pose. Although the method's generalizability is appealing, this estimation problem rapidly becomes underdetermined for an increased number of segments if no prior relationship between estimated B_0_ fields in different poses is exploited. To this end, a physics‐inspired B_0_ model has been used to connect the pose‐dependent B_0_ fields across poses.^19^, ^20^, ^21^ In Ref.^20^, this B_0_ model was successfully estimated from diffusion‐weighted magnitude images without the use of navigators.

Based on spatial and/or temporal B_0_ estimates, different solutions exist to correct for the induced B_0_ effects. Commonly, shimming is used to achieve more homogeneous B_0_ fields before (static shimming)^22^, ^23^ and/or during acquisition (real‐time shimming).^15^, ^16^ ^,^ ^24^ However, produced shim B_0_ fields generally lack spatial resolution to fully compensate the localized susceptibility‐induced B_0_ fields. Furthermore, real‐time shimming is often limited by temporal resolution because shim updates rely on B_0_ estimates from navigators, which are often time consuming to acquire and might be restricted by hardware constraints.^15^, ^16^ Additionally, post‐processing methods have been used extensively to correct B_0_ effects^25^, ^26^, ^27^, ^28^ but are limited to specific applications and/or sequences (mostly echo planar imaging (EPI)) and are usually not compatible with sophisticated acquisition techniques. Lastly, generalized reconstruction algorithms allow for the incorporation of the B_0_ variations in the forward model to remove artifacts.^29^ This has been successfully applied for intrascan motion correction by using navigator‐based B_0_ estimates.^7^ As mentioned before, the work in Ref.^20^ showed that a pose‐dependent B_0_ model can be estimated from diffusion‐weighted magnitude images. However, this was possible because the large number of diffusion‐weighted acquisitions (up to 65) created a highly overdetermined problem. This is substantially different from volumetric anatomical scans that generally consist of a single acquisition. To the best of our knowledge, a purely data‐driven intrascan motion‐corrected reconstruction for volumetric anatomical imaging that incorporates pose‐dependent B_0_ fields at high temporal resolution has not been reported yet.

This work aims to extend the aligned SENSE motion‐correction framework for volumetric anatomical imaging by considering pose‐dependent B_0_ variations in the head. First, the aligned SENSE forward model used to describe the MRI signal was extended to account for B_0_ variability. Next, a data‐driven approach was developed to estimate these B_0_ variations during retrospective motion correction. A compact physics‐inspired B_0_ model is used to model pose‐dependent B_0_ variations which, together with DISORDER sampling patterns, enables a purely data‐driven retrospective motion correction at 7 T, which is shown to improve the quality of the reconstructed images.

## 2 THEORY

### 2.1 Aligned SENSE motion correction

For volumetric encoding using array receiver coils and Cartesian sampling, the aligned SENSE framework^4^ achieves motion correction by dividing k‐space profiles (readouts) into temporal segments and allowing each segment to have a distinct motion state that can be estimated. This allows high (subsecond) temporal motion correction because parallel imaging provides an overdetermined problem to solve. The generalized reconstruction with rigid motion correction for parallel imaging can be formulated as an inverse problem in matrix form:

(1) $$\left(\hat{x},\hat{z_{n}}\right)=\underset{x,z_{n}}{\text{argmin}}\sum_{n}{\left|\left|A_{n}\mathrm{FS}T_{z_{n}}x-y_{n}\right|\right|}_{2}^{2},$$

where x is the volumetric image to be reconstructed; S the sensitivity profiles of the C‐element coil receiver array; and F the discrete Fourier transform. For each segment n, $T_{z_{n}}$ is the rigid motion operator with motion parameters $z_{n}$, $A_{n}$ the sampling mask, and $y_{n}\;$ the measured data for all coil elements. The reconstruction problem consists of estimating a 3D volume of size V from $N=C\sum_{n}E_{n}$ samples of a discretized k‐space grid for which $E_{n}$ represent the number of samples within each segment. The structure of the operators in Equation 1 is adapted from Ref.^4^ and is as follows:

- $y_{n}$ is a $E_{n}C\times 1$ vector.
- $A_{n}$ is a $E_{n}\mathrm{C\times VC}$ block matrix comprising submatrices of size $E_{n}\times V$ in which each row contains 1 element with value 1 at the acquired k‐space location for that sample and 0 elsewhere.
- F is a VC×VC block diagonal matrix comprising of submatrices of size V×V representing 3D discrete Fourier transforms.
- S is a VC×V block matrix comprising diagonal submatrices of size V×V whose diagonal elements correspond to the spatial sensitivity of the corresponding coil element.
- $T_{z_{n}}$ is a unitary^30^ matrix of size V×V corresponding to the 3D rigid transformation modeling the motion at segment n. $z_{n}$ are the motion parameters representing 3 translations and 3 rotations.
- x is a Vx1 vector.

### 2.2 Pose‐dependent B_0_ model

The total polarizing magnetic field in the scanner is a superposition of the B_0_ fields produced by the main magnet $B_{0,\text{magnet}}\left(r\right)\;$, the gradient system $B_{0,\mathrm{grad}}\left(r\right),$ and by other sources $B_{0,\text{other}}\left(r\right)\;(e.g.,$ magnet inhomogeneity, shims, susceptibility). Whereas $B_{0,\text{magnet}}(r)$ and $B_{0,\mathrm{grad}}\left(r\right)$ are intentionally produced respectively for tissue polarization and spatial encoding , $B_{0,\text{other}}\left(r\right)\;$ are often undesirable and will be referred to as $B_{0}\left(r\right)$ for the remainder of this work. For idealized spoiled gradient echo sequences, $\;B_{0}\left(r\right)$ induced frequencies ($\omega \left(r\right)=\gamma B_{0}\left(r\right)$, where γ is the gyromagnetic ratio) add a phase structure to the object's transverse magnetization (image)^31^:

(2) $$x\left(r\right)\to e^{-i2\pi \omega \left(r\right)\mathrm{TE}}x\left(r\right),$$

where TE is the echo time (TE) at which the center of k‐space in the readout direction is acquired. In matrix notation, this corresponds to a V×V diagonal matrix P with the exponential phase term exp(−i2πω(r)TE) on the diagonal elements that left multiplies the image : x→Px. In conventional image reconstruction, Px is the (complex) image to be reconstructed. When $B_{0}\left(r\right)$ dynamically changes with each segment, the phase term P becomes segment‐dependent, namely $P\left(\omega_{n}\left(r\right)\right)$, and Px can no longer be regarded as 1 entity. Instead, the segment‐dependent phase term must be explicitly included in the reconstruction formulation:

(3) $$\left(\hat{x},\hat{z_{n}},\hat{\omega_{n}}\right)=\underset{x,z_{n},\omega_{n}}{\text{argmin}}\sum_{n}{\left|\left|A_{n}\mathrm{FS}T_{z_{n}}P\left(\omega_{n}\right)x-y_{n}\right|\right|}_{2}^{2},$$

where $\omega_{n}$ represents the vector notation of the B_0_‐induced frequency $\omega_{n}\left(r\right)$ in segment n. Because P left multiplies x, $\omega_{n}$ is defined in the head co‐ordinate frame, which will be useful later when deriving a pose‐dependent B_0_ model. Because this work aims to perform data‐driven motion correction, $\omega_{n}$ in Equation 3 is jointly estimated together with the image and motion parameters. However, estimating a volumetric voxel‐based representation of the spatially and temporally varying $B_{0}$ at every shot would make the reconstruction underdetermined. Therefore, a more compact $\omega_{n}$ representation is needed. We resort to a first‐order Taylor expansion (with respect to the motion parameters) of the physics‐inspired model describing susceptibility‐induced B_0_ fields^10^:

(4) $$\omega_{n}\left(r\right)=d_{\text{pitch}}\left(r\right)\theta_{n,\text{pitch}}+d_{\text{roll}}\left(r\right)\theta_{n,\text{roll}},$$

where $d_{\text{pitch}}\left(r\right)$ and $d_{\text{roll}}\left(r\right)$ are the linear coefficient (LC) maps in pitch (around left–right (LR) axis) and roll (around posterior‐anterior axis) rotation angles $\theta_{n,\text{pitch}}$ and $\theta_{n,\text{roll}}$ (with $\theta_{n}\in z_{n}$) at shot n. A full derivation of Equation 4 is provided in Supporting Information Section 1. $d_{\text{pitch}}\left(r\right)$ and $d_{\text{roll}}\left(r\right)$ are defined in the head co‐ordinate frame and scale with $B_{0,\text{magnet}}\left(r\right)$. Note that a 0^th^ order term is missing in Equation 4 because this pose‐independent term is implicitly included in the complex image x (see Equation 2). Inserting Equation 4 into Equation 3 results in the following reconstruction problem:

(5) $$\left(\hat{x},\hat{z_{n}},\hat{d}\right)=\underset{x,z_{n},d}{\text{argmin}}\sum_{n}{\left|\left|A_{n}\mathrm{FS}T_{z_{n}}P_{\theta_{n}}\left(d\right)x-y_{n}\right|\right|}_{2}^{2},$$

with d representing $d_{\text{pitch}}\left(r\right)$ and $d_{\text{roll}}\left(r\right)$ in vector notation: $d=\left[d_{\text{pitch}};d_{\text{roll}}\right]$. Instead of estimating $\omega_{n}$ for every shot in Equation 3, we now only estimate d, which consists of 2 additional volumes as d is voxelized in this work.

Extending on the optimization approach proposed in the aligned SENSE reconstruction, problem 5 is tackled by iteratively updating x, $z_{n}$, and d:

(6a) $$x^{i+1}=\underset{x}{\text{argmin}}\sum_{n}{\left|\left|A_{n}\mathrm{FS}T_{z_{n}^{i}}P_{\theta_{n}}\left(d^{i}\right)x-y_{n}\right|\right|}_{2}^{2}$$

(6b) $${z_{n}}^{i+1}=\underset{z_{n}}{\text{argmin}}\sum_{n}{\left|\left|A_{n}\mathrm{FS}T_{z_{n}}P_{\theta_{n}}\left(d^{i}\right)x^{i+1}-y_{n}\right|\right|}_{2}^{2}$$

(6c) $$d^{i+1}=\underset{d}{\text{argmin}}\sum_{n}{\left|\left|A_{n}\mathrm{FS}T_{z_{n}^{i+1}}P_{\theta_{n}}\left(d\right)x^{i+1}-y_{n}\right|\right|}_{2}^{2}.$$

Problems 6a, 6b, and 6c can be solved with respectively the conjugate gradient, Levenberg–Marquardt, and gradient descent algorithms. The expression and mathematical derivation of the gradient descent update used in Equation 6c is described in Appendix A. As identified previously,^32^ we note that problem 6c is highly nonconvex because the exponential term in $P_{\theta_{n}}$ has periodic saddle points causing phase‐wrapping ambiguities. A phase‐wrapping stabilizer was added to the optimization to reduce these ambiguities (see Supporting Information Section 2).

## 3 METHODS

### 3.1 Data acquisition

Volumetric SPGR scans were acquired on 2 consented adult healthy volunteer subjects on a 7 T scanner (MAGNETOM Terra, Siemens Healthcare, Erlangen, Germany) with a 1Tx coil (Nova Medical) and a 3 T scanner (ACHIEVA TX, Philips Medical Systems, Best, The Netherlands) using a C = 32‐element adult head coil array. Sequence parameters are the same for both 3 T and 7 T acquisitions: TE = 5 ms, repetition time TR = 10 ms, flip angle = 7, readout along IS dimension to exclude the neck region from rigid motion estimation^5^ and the random‐checkered DISORDER sampling pattern in the phase‐encoding dimensions with subsecond shot durations. No repeats or acceleration were applied to concentrate on the effect of the proposed model. An elliptical shutter was used in both phase‐encoding dimensions.

Acquired SPGR scans are used in 2 experiments. Experiment 1 (pose experiment) investigates the pose‐dependent nature of B_0_ fields by acquiring 8 scans in which the subject held a single and different pose for each scan. This experiment was conducted on the first subject on both a 3 T and 7 T scanner. Additional sequence parameters were at 3 T, $\;1.50\times 1.741.74\;mm^{3}$ resolution, field of view (FOV) = 258 × 240 × 250 $\;mm^{3}$ (inferior‐superior (IS)/AP/LR), acquisition time = 2 min 35 s; and at 7 T, $1.51\times 1.51\times 1.50\;mm^{3}$ resolution, FOV = 220 × 220 × 264 $\;mm^{3}$ ( IS/AP/LR), acquisition time = 3 min 28 s.

Experiment 2 (motion experiment) consisted of a controlled motion experiment on the 7 T scanner where both subjects were asked to change pose every 20 s. This was repeated for low, medium, and high instructed motion levels, corresponding with respective rotation angles of approximately ±1°, ±3°, and ±8°. Rotation angles vary between both subjects (see Results) because precise motion cannot be enforced. Additional sequence parameters for subject 1 were $:1.47\times 1.47\times 1.5\;mm^{3}$ resolution, FOV = 220 × 200 × 240 $\;mm^{3}$ (IS/AP/LR), acquisition time = 2 min 57 s; and for subject 2, $1.55\times 1.55\times 1.49\;mm^{3}$ resolution, FOV = 229 × 272 × 214 $\;mm^{3}$ (IS/AP/LR), acquisition time = 3 min 25 s.

For each scan session on both scanners, coil array sensitivity profiles used in the reconstructions were estimated from a separate low‐resolution ($6\times 6\times 6\;mm^{3}$) reference scan using a custom implementation of the ESPIRiT algorithm.^33^

### 3.2 Pose‐dependent B_0_ model

Because individual acquisitions in experiment 1 (pose experiment) contain negligible motion levels, all 8 scans for both 3 T and 7 T data are reconstructed without considering motion or B_0_, corresponding to a SENSE reconstruction. Because the B_0_ model in Equation 4 is defined in the head frame, the 8 scans from each subexperiment at 3 T and 7 T are co‐registered to the corresponding reference volumes (defined as the first of 8 acquired volumes, positioned in the FOV center) using an in‐house built rigid registration with 6 degrees of freedom (3 translations and 3 rotations). Based on the assumption that the induced B_0_ field acts as an additional phase term in the complex image (Equation 2), the induced B_0_ fields are obtained by unwrapping^34^ the phase difference φ between each scan relative to the reference scan and converting it to frequency: ω=φ/2πTE. Finally, LC maps $d_{\text{pitch}}$ and $d_{\text{roll}}$ are fitted by performing a voxelized least‐squares fit on Equation 4 with $\theta_{n,\text{pitch}}$ and $\theta_{n,\text{roll}}$ obtained from the rigid registration. After $d_{\text{pitch}}$ and $d_{\text{roll}}$ are fitted for each of the sub‐experiments at 3 T and 7 T, they are co‐registered for anatomical consistency.

### 3.3 Synthetic experiments

We built a synthetic dataset from the 7 T data in experiment 1: the reference image, estimated coil array sensitivity profiles, and the LC maps $d_{\text{pitch}}$ and $d_{\text{roll}}$ fitted from the acquired data serve as ground truth (GT) values. Simulations were performed to assess the effect of pose‐dependent B_0_ fields on the aligned SENSE motion correction and to investigate the ability to estimate the $\omega_{n}$ model described in Equation 4. The forward model in the presence of rigid motion and motion‐induced B_0_ fields is applied to generate synthetically corrupted data. Synthesized k‐space is divided into 128 shots using the DISORDER sampling. Each shot contains a different motion state. Motion parameters are designed to mimic a realistic interleaved motion trace: 13 different poses are generated (128/13 shots per pose) by randomly sampling translation and rotation parameters from a uniform distribution of respectively [−5 5] mm and [−7.5 7.5] °. Within each pose, a slow drift back toward the neutral position was added to mimic observed motion traces in vivo (see Results). Synthesized motion traces are shown in Supporting Information Figure S1. Noise was added to the synthesized k‐space corresponding to an estimated mean SNR of 30 dB for reconstructions in the absence of motion or B_0_ variations. To assess the effect of noise on the optimization, the same experiment was repeated without adding noise.

The synthesized k‐space is reconstructed in different ways: “uncorrected” without motion or B_0_ modeling (corresponding to classical SENSE), “motion corrected” with only motion correction (corresponding to aligned SENSE), and the proposed “motion + B_0_ corrected” with additional correction for B_0_ variations. Finally, to investigate whether residuals in the motion + B_0_‐corrected reconstruction arise from the suboptimal convergence or by the addition of B_0_ to the forward model, the synthesized k‐space is reconstructed when both GT motion and GT B_0_ parameters are provided and only the image is reconstructed (referred to as “(motion + B_0_) provided”). This is then compared to the motion‐free case in which no motion (and hence B_0_) is applied when synthesizing k‐space, which is reconstructed without correction. The last 2 experiments are referred to as the *reference reconstructions* because they require GT values to be known and cannot be used in vivo therefore serving as the upper limit of the attainable image quality.

For motion‐corrected and motion + B_0_‐corrected reconstructions, the alternating optimization ran for 45 and 15 outer iterations, respectively, for the levels at half ($3\times 3\times 3\;mm^{3})$ and full ($1.5\times 1.5\times 1.5\;mm^{3})$ resolution. Reconstructed images x are evaluated by computing the $\mathrm{SNR}=10\log_{10}\left({\bar{\left|x\right|}}^{2}/{\bar{\left|x-x_{\mathrm{GT}}\right|}}^{2}\right)$ with respect to the GT image $x_{\mathrm{GT}}$.

### 3.4 In vivo reconstructions

The in vivo acquisitions from experiment 2 are reconstructed using the uncorrected, motion‐corrected, and the proposed motion + B_0_‐corrected reconstruction. For comparison, reconstructed complex images are registered to a motion‐free reference scan and errors are evaluated by computing the Structural Similarity Index Measure,^35^ Mutual Information,^36^ SNR, and Artifact Power^37^ with respect to this reference image. A MatLab (MathWorks, Natick, MA) implementation to reproduce all experiments is made available at https://github.com/mriphysics/B0InformedDISORDER/releases/tag/1.0.0. Implementation details for the reconstructions are reported in Supporting Information Section 2. A second‐order Taylor expansion of the B_0_ model was implemented as well but did not improve reconstructions (results and derivations not shown) and therefore will not be elaborated on. Reconstructions are performed on a 20(40) × Intel(R) Xeon(R) Silver 4210 2.20 GHz CPU, 251 GB RAM, 32GB NVIDIA Tesla V100 GPU (Nvidia, Sanata Clara, CA, USA). The longest computation time for a 1.5 mm^3^ isotropic motion +B_0_‐corrected reconstruction was 22 min.

## 4 RESULTS

### 4.1 Pose‐dependent B_0_ model

**FIGURE 1:**
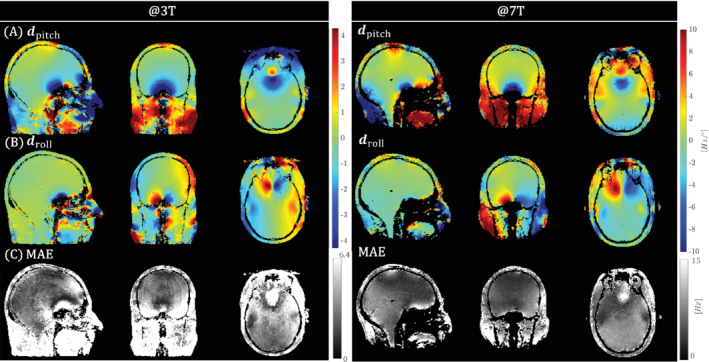
In vivo experiment 1 (pose). LC maps $d_{\text{pitch}}\;$(A) and $d_{\text{roll}}$ (B) at 3 T and 7 T, together with the MAE (C). LC maps were separately fitted on the induced B_0_ fields across the acquired poses for 3 T and 7 T data. Fitted 3 T and 7 T LC maps are registered to allow anatomical comparison. Note that the display range differs between 3 T and 7 T, accounting for the field strength dependence of the B_0_ model. For visual purposes, tissue is extracted by thresholding the corresponding magnitude images. LC, linear coefficient; MAE, mean absolute error; T, Tesla

Pose information from the image registration for all motion‐free scans in experiment 1 is listed in Table 1. Scans in the 3 T experiment cover a larger pose range (up to 15°) compared to the 7 T experiment (up to 10°). Figure 1A,B show the fitted $d_{\text{pitch}}$ and $d_{\text{roll}}$ LC maps, with the mean absolute error (MAE) shown in Figure 1C. Note that the color bars in Figure 1A,1B are scaled to make the maps field strength–independent. When rescaled to account for B_0_,_magnet_, LC maps at both field strengths show very good similarity. In all LC maps, the symmetry with respect to both roll and pitch rotation axis can be seen, and opposite signs occur at opposite sides. Areas that show large absolute values for $d_{\text{pitch}}$ or $d_{\text{roll}}$ are situated close to air−tissue boundaries (especially above the sinuses and the ear canals), which is a known feature of susceptibility‐induced B_0_ fields. In those areas, the MAE is bigger compared to areas in the middle of the brain. Lastly, we note that the MAE map at 3 T has an overall higher value compared to the, 7T MAE map.

**TABLE 1:** In vivo experiment 1 (pose): Pose information after image registration for the different (single pose) scans relative to the first one

		Scan 1	Scan 2	Scan 3	Scan 4	Scan 5	Scan 6	Scan 7	Scan 8
@3 T	Pitch [°]	0	4.80	−1.21	−9.05	14.22	−13.28	8.52	−9.28
	Roll [°]	0	4.55	15.63	15.39	2.91	1.70	−14.26	−16.36
	Yaw [°]	0	−5.67	−4.82	−5.40	−3.60	−2.19	0.38	2.74
@7 T	Pitch [°]	0	3.76	4.02	−4.47	3.22	3.41	0.56	−6.41
	Roll [°]	0	0.55	7.37	2.34	2.46	−6.55	−6.69	−10.77
	Yaw [°]	0	−1.21	−1.52	1.89	0.76	1.54	−1.42	−0.48

*Note*: The first scan was obtained in the equilibrium position (read: FOV centre). Pose information is defined with respect to the isocentre using a pitch‐roll‐yaw Euler‐angles convention. Translation parameters are not included because they do not contribute to the pose dependent B_0_ model. For the same reason, yaw parameters are grayed out.

### 4.2 Simulations

**FIGURE 2:**
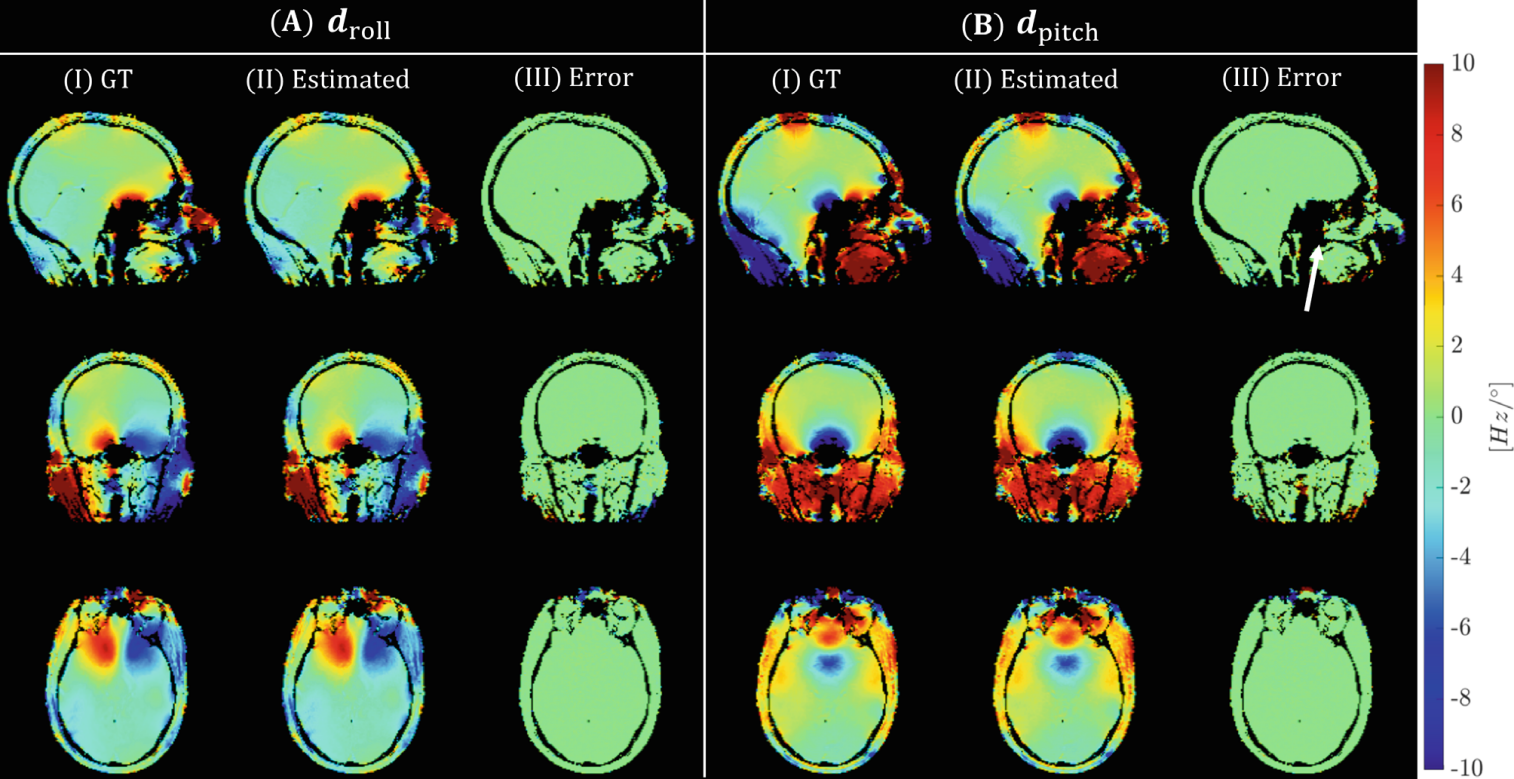
Simulations. Reconstruction performance regarding the LC maps $\;d_{\text{roll}}$ (A) and $\;d_{\text{pitch}}$ (B). For each LC map, the GT used to generate simulated data (I) is compared to the estimated LC maps (II), together with corresponding residuals (III). Error maps are small inside the brain with localized errors outside (white arrow). For visual purposes, tissue is extracted by thresholding the corresponding magnitude images. GT, ground truth

**FIGURE 3:**
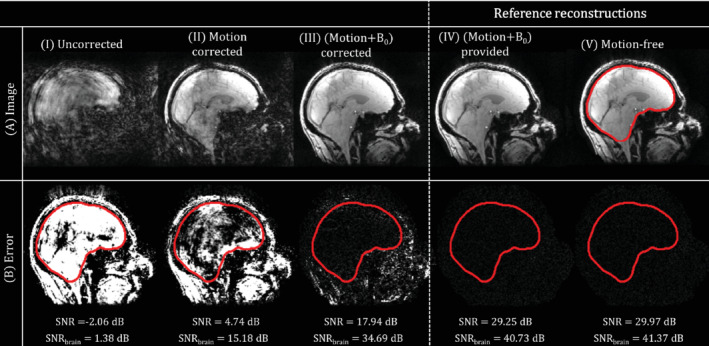
Simulations. Reconstructed images (A) and the corresponding residuals with respect to the GT (B) for the uncorrected (I), motion‐corrected (II), and (motion+B_0_)‐corrected reconstruction (III) compared to the reference reconstructions (IV‐V). SNR values are shown for the whole FOV and the brain only (delineated in red). Note that the displayed error range in (B) is 10% of the display range in (A)

Results of the simulated experiments are shown in Figures 2 and 3. Figures 2A.I,2B.I show the LC maps $d_{\text{roll}}$ or $d_{\text{pitch}}$ obtained from the motion +B_0_‐corrected reconstruction of the synthesized k‐space. The GT LC maps used for data synthesis and the respective error are shown in the columns to the right (Figures 2A.II–III,2B.II–III). Residuals are very small inside the brain, with very localized errors outside the brain (e.g., at the arrow in Figure 2B.III). These localized errors outside the brain do not come from the noise added to the synthesized k‐space because similar errors appear in the noise‐free simulations (Figure S2).

Figure 3A,2B show the reconstructed anatomical images and their corresponding errors compared to the GT image used for data synthesis. The SNR is shown at the bottom of Figure 3B for both the whole FOV as well as for the brain region only (delineated in red). The uncorrected (Figure 3A.I), motion‐corrected (Figure 3A.II), and the proposed motion + B_0_‐corrected reconstruction (Figure 3A.III) are compared to the reference reconstructions (Figure 3A.IV,V). The SNR in the motion‐free experiment in Figure 3B.V is the expected noise level (∼30 dB). Because the other reconstructions have lower SNR values, this is indeed the upper limit for the attainable image quality. Reduced errors are observed when increasing the model complexity (from left to right). When not accounting for motion (Figure 3A.I), the image is highly corrupted. Correcting for motion (Figure 3A.II) clearly improves image quality and SNR; however, it still contains artifacts such as signal loss around the sinuses as well as in overall signal contrast. Using the proposed motion + B_0_‐corrected reconstruction (Figure 3A.III) shows a further increase in SNR compared to the motion‐corrected reconstruction.

Residuals for the proposed motion + B_0_‐corrected reconstruction (Figure 3B.III) are mostly situated outside the brain. This observation is confirmed when comparing the SNR to the reference reconstructions: the SNR difference is smaller for the brain region (6.04dB) compared to the whole FOV (11.31dB). The location of these residuals corresponds to those in the reconstructed LC maps (see Figure 2), showing the coupling of both LC maps and image reconstruction. These localized errors outside the brain appear in the noise‐free simulation as well (Figure S3), consistent with the earlier observation in Figure 2.

Finally, the SNR difference between the reference reconstructions (Figure 3B.IV,V) is very small compared to the observed SNR in the other reconstructions. This shows that adding pose‐dependent B_0_ to the signal model does not highly deteriorate the conditioning of the reconstruction.

### 4.3 In vivo motion experiments

**FIGURE 4:**
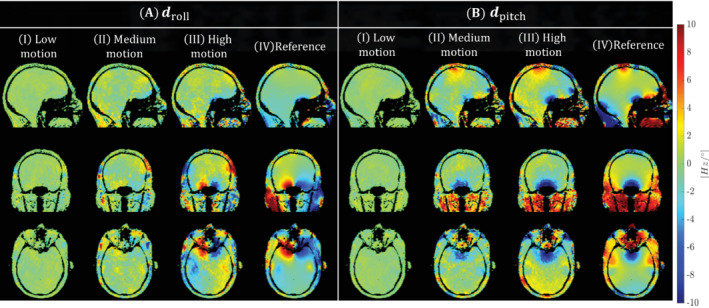
In vivo experiment 2 (motion). Estimated LC maps $\;d_{\text{roll}}$ (A) and $\;d_{\text{pitch}}$ (B) for subject 1 in the different controlled motion experiments (I–III). The LC maps obtained from experiment 1 (pose experiment) are shown as a reference (IV) for both $\;d_{\text{roll}}$ and $\;d_{\text{pitch}}$. Although the estimated and reference LC maps should not perfectly match in theory, they provide an indication of the quality of reconstruction. For visual purposes, tissue is extracted by thresholding the corresponding magnitude images

**FIGURE 5:**
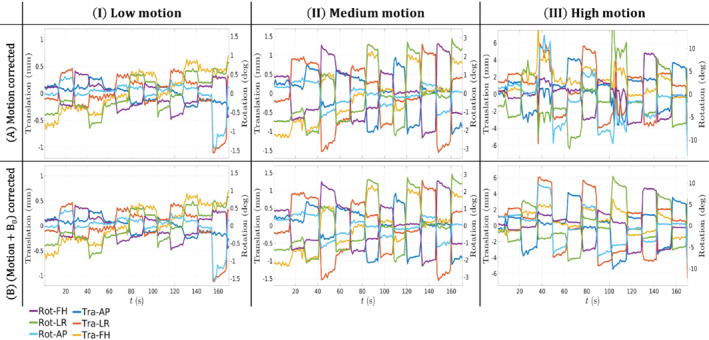
In vivo experiment 2 (motion). (I–III) Estimated motion traces for subject 1 in the different controlled motion experiments for the (A) motion‐corrected and (B) motion + B_0_‐corrected reconstruction. The horizontal time axis is removed from (A) to improve clarity. Labels for translation (Tr) are defined for the LR, AP, and FH directions. Labels for Rot are defined for rotation around the LR, AP, and FH axis, respectively pitch, roll, and yaw rotation. AP, anterior–posterior; FH, foot‐head; LR, left–right; Rot, rotation

Figure 4A,4B respectively show the estimated LC maps $d_{\text{pitch}}$ and $d_{\text{roll}}$ in the controlled motion experiments (experiment 2) for subject 1 for each of the motion levels (Figure 4A.I‐III,B.I‐III). LC maps fitted from the pose experiment (experiment 1) are added as a reference (Figure 4A.IV,4B.IV). For low motion levels (Figure 4A,4B.I), LC maps show no resemblance with the reference LC maps, whereas the high‐motion case (Figure 4A,4B.III) shows clear agreement. This is caused by the nature of the pose‐dependent B_0_ fields: when little motion is present, B_0_ variations are small and have limited contribution to the signal. Figure S4 shows the estimated LC maps for subject 2, where the same observations hold. For the medium motion experiment (Figure 4A,4B.II), LC estimates seem more accurate for $d_{\text{pitch}}$ than for $d_{\text{roll}}$ because associated motion parameters (Figure 5A.II) show a larger range for $\theta_{\text{pitch}}$ (green curve) than $\theta_{\text{roll}}$ (cyan curve), a usual pattern of motion in the scanner. Other motion parameters for subject 1 in experiment 2 are shown in Figure 5 (correspondingly in Figure S5 for subject 2) for both the motion‐corrected (Figure 5A) and proposed reconstruction (Figure 5B). For the low motion experiment (Figure 5A,B.I), there is no noticeable effect in the motion traces when including the B_0_ model. However, at higher motion levels (Figure 5A,B.III), incorporating the motion‐induced B_0_ fields results in motion traces with lower fluctuations. Because the subject was asked to hold pose during interleaved timings, motion traces from the proposed reconstruction (Figure 5B.III) are assumed to be more realistic. This improved smoothness of the motion traces is also observed in the medium motion experiment but to a lesser extent.

**FIGURE 6:**
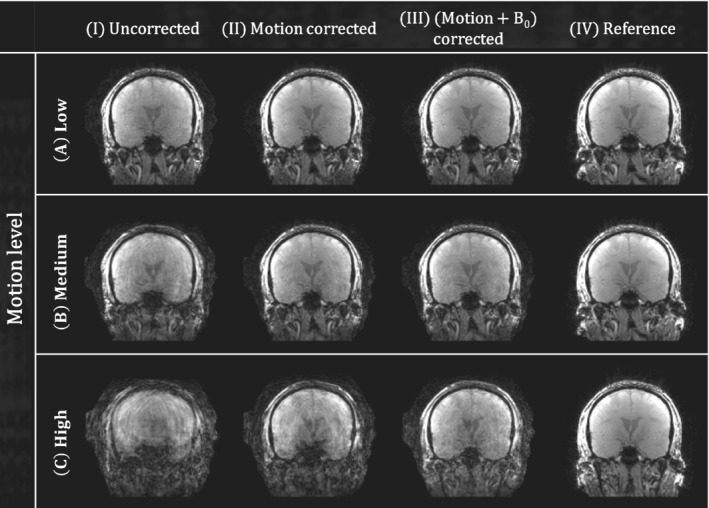
In vivo experiment 2 (motion). Coronal view of the reconstructed images for subject 1. The different subexperiments are shown in the rows (A–C), and the different reconstruction methods are shown in the columns (I–III). A motion‐free scan from experiment 1 is added as a reference (IV)

A coronal view of the reconstructed images for subject 1 in experiment 2 is shown in Figure 6 (correspondingly in Figure S6 for subject 2). The inclusion of the pose‐dependent B_0_ model for low and medium motion levels (Figure 6A.III,6B.III) shows little improvement compared to the motion‐corrected reconstruction (Figure 6A.II,6B.II). This is consistent with the observation in the reconstructed LC maps and motion parameters: when there is little motion, the B_0_ changes have a small effect and therefore do not contribute much to the reconstruction. On the contrary, clear improvement is observed at higher motion levels (Figure 6C.III). Similarity metrics for the different reconstructions with respect to the reference motion‐free image are shown in Table 2. For all experiments, improved similarity is obtained when modeling motion in the forward model. Modeling the pose‐dependent B_0_ fields further improves the similarity metrics, although to a lesser extent. Finally, similarity metrics for the proposed reconstruction improve when motion levels decrease. This shows that the proposed motion and B_0_ corrections for the high motion experiment are not able to reach the same image quality as for the low‐motion experiments.

**TABLE 2:** In vivo experiment 2 (motion): Similarity metrics for the reconstructed images with respect to the corresponding motion‐free reference image

		Low Motion				Medium Motion				High Motion			
		SSIM [10^−1^]	MI	SNR	AP [10^−1^]	SSIM [10^−1^]	MI	SNR	AP [10^−1^]	SSIM [10^−1^]	MI	SNR	AP [10^−1^]
Subject 1	Uncorrected	3.15	1.42	7.83	1.29	2.52	1.32	6.17	1.63	1.10	1.09	1.52	3.12
	Motion‐corrected	3.28	1.47	8.15	1.27	2.92	1.39	7.54	1.33	1.74	1.18	3.82	2.35
	(Motion + B_0_)‐corrected	3.28	1.47	8.15	1.27	2.95	1.41	7.84	1.28	2.00	1.22	6.23	1.78
Subject 2	Uncorrected	5.09	2.26	18.10	0.15	4.57	2.01	15.56	0.26	1.90	1.39	5.70	1.75
	Motion‐corrected	5.22	2.37	18.73	0.13	4.83	2.14	17.15	0.19	2.85	1.54	8.62	1.10
	(Motion + B_0_)‐corrected	5.22	2.37	18.73	0.13	4.84	2.15	17.17	0.19	3.02	1.67	10.62	0.79

Abbreviations: AP, Artefact Power; MI, Mutual Information; SSIM, Structural Similarity Index Measure.

**FIGURE 7:**
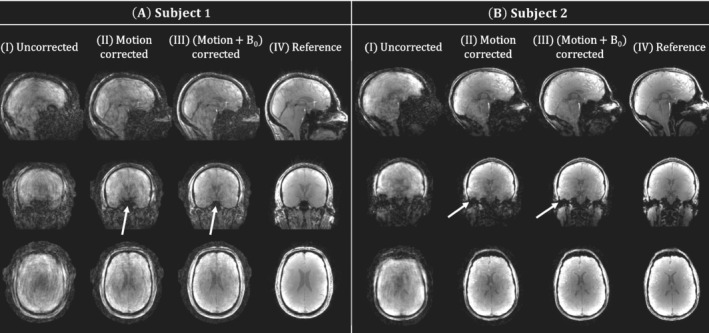
In vivo experiment 2 (motion). Sagittal (top), coronal (middle), and transversal (bottom) view of experiment 2C (highest motion level) for both subjects and with the different reconstruction methods. For subject 1, a motion‐free scan from experiment 1 is added as a reference. For subject 2, a motion‐free scan from the same scan session is added as a reference. The proposed motion + B_0_‐corrected reconstruction improves overall contrast (e.g., transversal view) and recovers signal (arrows)

All reconstructions for the high motion experiment are shown in Figure 7 for both subjects. Reduced image artifacts are observed for the proposed reconstruction (Figure 7A‐7B.III) compared to motion correction (Figure 7A,7B.II). The reduced artifacts emerge as recovered signal near air–tissue interfaces (white arrows) and improved contrast (e.g., around the ventricles).

## 5 DISCUSSION

We have presented a data‐driven retrospective intrascan motion‐correction algorithm for volumetric anatomical imaging that accounts for pose‐dependent B_0_. Based on the underlying physics, pose‐dependent B_0_ are modeled to scale linearly with spatially varying LC maps in pitch and roll rotation angles. The use of a physics‐inspired B_0_ model enabled the data‐driven approach and provides an alternative to the use of B_0_ navigators previously presented for motion correction.^7^ Previous work exploiting this physics‐inspired B_0_ model for motion correction either calibrated this model before the examination^19^ or had an abundance of acquisitions available to estimate the model.^20^ In our work, we enable the use of this model in intrascan motion correction at high temporal resolution. Conducted simulations have shown that the B_0_ model can be estimated and can improve the final image quality. In vivo tolerance to motion using the proposed reconstruction has been demonstrated using a controlled experiment, with improved image quality in scans where strong motion was present throughout the examination.

LC maps obtained in the motion‐free experiments show localized features near the nasal cavities and the ear canals. In those areas, observed MAE are higher. This is expected to be caused by imaging limitations in those areas (distortion in the readout direction) and associated registration performance. The increased MAE in the 3 T data compared to the 7 T data is expected because a Taylor approximation should model the underlying data better for small pose deviations (7 T) than for larger pose deviations (3 T). Including other B_0_ fields with a pose‐dependent nature (e.g., body susceptibility or shims, which are fixed in the scanner co‐ordinates) did not show improvements; therefore, we further need to analyze how to successfully incorporate them. Higher‐order Taylor expansions of the susceptibility‐induced B_0_ fields were considered but will have small effects on the B_0_ signal for considered motion levels, and they would further increase the number of parameters to estimate, making this approach difficult to deal with. The ranges of LC maps at 7 T are in the order −10 to 10 Hz/degree, which is in accordance with values reported in literature.^20^ ^,^ ^38^ Furthermore, excellent agreement is observed between both 3 T and 7 T LC maps with similar features and dynamic ranges that scale with field strength ($B_{0,\text{magnet}}$). This confirms that the applicability of the pose‐dependent B_0_ model is not limited to 7 T MRI and can also be applied at other field strengths. However, for lower field strengths, these effects will become less substantial, and much larger motion levels are needed to have the same effect on the signal. This also depends on the sequence because the works by Hutton et al.^19^ and Andersson et al.^20^ already show significant distortion effects of the pose‐dependent B_0_ fields at 3 T for EPI.

Simulations show that we can jointly estimate the LC maps with the image and motion parameters in the presence of noise. LC maps and the image are reconstructed well with minor residuals outside the brain. Results showed that observed residuals did not arise from the added noise but from the altered optimisation landscape, making noise not the limiting factor in this joint reconstruction.

Improved reconstructions are also observed in vivo for the proposed motion + B_0_‐corrected reconstruction at increased motion levels (−10 to 10 degrees of $\theta_{\text{pitch}}$ or $\theta_{\text{roll}}$). The quality of the reconstructed LC maps is poorer than in Andersson et al.^20^ and can be explained by the limited acquired data. Additionally, increased B_0_ sensitivity due to distortion might also be beneficial for the B_0_ estimation in 20. For small motion levels, the original motion correction provides satisfactory results consistent with previous work showing successful application of DISORDER for small motion levels at 7 T.^6^ In those cases, the addition of the pose‐dependent B_0_ model has negligible effect on the signal model (θ≅0). Additionally, performance should be evaluated for other types of motion. For example, when only a few shots are corrupted with motion, this method will not perform optimally because a range of poses are required to result in reliable LC maps. In case of suboptimal performance, methods using B_0_ navigators^7^ or pre‐calibrated LC maps^19^ could result in better reconstructions at the expense of decreased scanning efficiency. Pretrained regularizers (using physics‐inspired models or machine learning) could further improve the reconstruction performance.

The proposed method at high and medium motion levels does not attain the same image quality as in the previously proposed motion + B_0_‐corrected reconstruction by Liu et al.^7^ However, the latter required navigators to measure motion and B_0_, whereas our method relies on data‐driven estimates. Furthermore, motion levels considered in this study are larger than in,^7^ which might have additional implications for the reconstruction: First, gaps in k‐space are more likely to be created for increased motion levels. Second, the proposed signal model might be inaccurate as some of the approximations valid at lower field strengths might not be satisfied at 7 T and could be magnified at higher motion levels. For example, transmit (B_1_^+^) and receiver (B_1_^−^) coil sensitivity profiles might change with pose due to coil loading, which would require modifying the proposed signal model.^39^ Because no evidence is presented for this yet, further analysis is needed. Next, this work modeled the effect of varying B_0_ as pose‐dependent phase structures to an unchanged anatomical complex image. However, changes in B_0_ will also affect the spin behavior. This effect has been investigated in a preliminary experiment using the extended phase graph formalism^40^ and did not show big effects for motion traces considered in this study (transient error up to 10% in signal intensity after abrupt pose changes). This is consistent with simulations conducted by Sulikowska et al.^21^ However, for continuous motion throughout the acquisition, the SPGR signal in extended phase graph simulations show strong variations over time, breaking the assumption of an unchanged object moving in the scanner. Therefore, a more thorough analysis should assess the effect of different types of motion on a range of clinical sequences to reveal the limits of motion correction at 7 T.

## 6 CONCLUSION

We have presented a retrospective framework for motion‐tolerant structural 3D brain imaging by incorporating pose‐dependent B_0_ fields in the forward model for Cartesian SPGR sequences at 7 T. By deploying a physics‐inspired B_0_ model, the number of parameters needed to characterize pose‐dependent B_0_ fields is limited, enabling a purely data‐driven reconstruction that jointly estimates the image, motion, and B_0_ variations and that improves reconstruction quality. Effects of the pose‐dependent B_0_ fields at increased motion levels and the ability to correct for them have been demonstrated in simulations and in vivo.

## CONFLICT OF INTEREST

Dr. Raphael Tomi‐Tricot is currently employed by Siemens Healthcare.

## Supporting information


**Figure S1**. Simulations. Estimated motion parameters from the synthesized k‐space with (A) and without (B) the addition of noise for the motion‐corrected and motion + B_0_‐corrected reconstruction. GT motion parameters are shown on the right. Labels for translation (Tr) are defined for the left–right (LR), anterior–posterior (AP) and foot‐head (FH) direction. Labels for rotation (Rot) are defined for rotation around the LR, AP and FH axis, respectively pitch, roll and yaw rotation
**Figure S2**. Simulations in the absence of noise. Reconstruction performance regarding the LC maps $d_{\text{roll}}$ (A) and $d_{\text{pitch}}$ (B). For each LC map, the GT used to generate simulated data (I) is compared to the estimated LC maps (II), together with corresponding residuals (III). Error maps are small inside the brain with localized errors outside (white arrow). For visual purposes, tissue is extracted by thresholding the corresponding magnitude images
**Figure S3**. Simulations in the absence of noise: Reconstructed images (A) and the corresponding residuals with respect to the GT (B) for the uncorrected (I), motion‐corrected (II) and motion+B_0_‐corrected reconstruction (III) compared to the reference reconstructions (IV–V). Note that the displayed error range in (B) is 10% of the display range in (A)
**Figure S4**. In‐vivo Experiment 2 (motion). Estimated LC maps $\;d_{\text{roll}}$ (A) and $d_{\text{pitch}}$ (B) for subject 2 in the different controlled motion experiments (I–III). For visual purposes, tissue is extracted by thresholding the corresponding magnitude images
**Figure S5**. In‐vivo Experiment 2 (motion). (I–III) Estimated motion traces for subject 2 in the different controlled motion experiments for the (A) motion‐corrected and (B) motion + B_0_‐corrected reconstruction. The horizontal time axis is removed from (A) to improve clarity. Labels for translation (Tr) are defined for the left–right (LR), anterior–posterior (AP) and foot‐head (FH) directions. Labels for rotation (Rot) are defined for rotation around the LR, AP and FH axis, respectively pitch, roll and yaw rotation
**Figure S6**. In‐vivo Experiment 2 (motion). Coronal view of the reconstructed images for subject 2. The different sub‐experiments are shown in the rows (A–C) and the different reconstruction methods are shown in the columns (I–III). A motion free scan from is added as a reference (IV)

## APPENDIX A

### A.1

GRADIENT DESCENT UPDATE FOR THE POSE‐DEPENDENT B_0_ MODEL

The optimisation problem in Equation 6c consists of estimating the LC maps $d_{\text{pitch}}$ and $d_{\text{roll}}$ from k‐space data:

(A1)
$$\hat{d}=\underset{d}{\text{argmin}}\sum_{n}{\left|\left|A_{n}\mathrm{FS}T_{z_{n}}P_{\theta_{n}}\left(d\right)x-y_{n}\right|\right|}_{2}^{2}=\underset{d}{\text{argmin}}L\left(d\right)$$

where L(d) the data‐consistency loss function in $d≜\left[d_{\text{pitch}};d_{\text{roll}}\right]$. Definitions of the other operators can be found in the main body of the text. The gradient of L(d) with respect to d can be expressed as follows:

(A2)
$$\nabla_{d}^{L}=2\sum_{n}R\left(v_{n}^{H}w_{n}\right)$$

where R extracts the real component and:

(A3a)
$$w_{n}=A_{n}\mathrm{FS}T_{z_{n}}P_{\theta_{n}}\left(d\right)x-y_{n}$$

(A3b)
$$\begin{array}{cc} v_{n}\; & \;=A_{n}\mathrm{FS}T_{z_{n}}\frac{\partial P_{\theta_{n}}\left(d\right)}{\partial d}D\left(x\right)\\ & =A_{n}\mathrm{FS}T_{z_{n}}\frac{\partial \left(e^{-i2\pi \omega_{n}\left(d\right)\mathrm{TE}}\right)}{\partial d}D\left(x\right) \end{array}$$

where D(x) is a diagonal matrix with the elements of x on the diagonal. By replacing $\omega_{n}\left(d\right)$ with the linear model from Equation 4, the derivatives in Equation A3b can be expressed separately for $d_{\text{pitch}}$ and $d_{\text{roll}}$:

(A4a)
$$\begin{array}{cc} \;\frac{\partial P_{\theta_{n}}(d)}{\partial d_{\text{pitch}}} & \;=\frac{\partial \left(e^{-i2\pi \left(\theta_{n,\;\text{pitch}}d_{\text{pitch}}+\theta_{n,\text{roll}}d_{\text{roll}}\right)\mathrm{TE}}\right)}{\partial d_{\text{pitch}}}\\ & \;=-i2\pi \theta_{n,\;\text{pitch}}\mathrm{TE}P_{\theta_{n}}(d) \end{array}$$

(A4b)
$$\begin{array}{cc} \;\frac{\partial P_{\theta_{n}}\left(d\right)}{\partial d_{\text{roll}}} & \;=\frac{\partial \left(e^{-i2\pi \left(\theta_{n,\;\text{pitch}}d_{\text{pitch}}+\theta_{n,\text{roll}}d_{\text{roll}}\right)\mathrm{TE}}\right)}{\partial d_{\text{roll}}}\\ & \;=-i2\pi \theta_{n,\;\text{roll}}\mathrm{TE}P_{\theta_{n}}\left(d\right) \end{array}$$

Which results in the gradients:

(A5a)
$$\begin{array}{cc} \nabla_{d_{\text{pitch}}}^{L} & \;=2\sum_{n}R\left(+i2\pi \theta_{n,\;\text{pitch}}\mathrm{TE}D\left(x^{H}\right)P_{\theta_{n}}^{H}\left(d\right)T_{z_{n}}^{H}A_{n}^{H}\right.\\ & \left.\left(A_{n}\mathrm{FS}T_{z_{n}}P_{\theta_{n}}\left(d\right)x-y_{n}\right)\right) \end{array}$$

(A5b)
$$\begin{array}{cc} \nabla_{d_{\text{roll}}}^{L} & \;=2\sum_{n}R\left(+i2\pi \theta_{n,\;\text{roll}}\mathrm{TE}D\left(x^{H}\right)P_{\theta_{n}}^{H}\left(d\right)T_{z_{n}}^{H}A_{n}^{H}\right.\\ & \left.\left(A_{n}\mathrm{FS}T_{z_{n}}P_{\theta_{n}}\left(d\right)x-y_{n}\right)\right) \end{array}$$

Where the symbol i is the imaginary number. By re‐arranging the gradients in 1 variable $\nabla_{d}^{L}≜\left[\nabla_{d_{\text{pitch}}}^{L};\nabla_{d_{\text{roll}}}^{L}\right]$, we can resort to a gradient descent update of the LC maps d:

(A6)
$$d^{i+1}=d^{i}-\lambda \nabla_{d}^{L}$$

where λ is the step size and i now represents the iteration number, consistent with Equation 6c.
