# Supplementary material for: Data‐driven motion‐corrected brain MRI incorporating pose‐dependent B0 fields
Source: Magn Reson Med. 2022 May 8;88(2):817–31. doi: 10.1002/mrm.29255 (PMC9324873; doi:10.1002/mrm.29255)
Supplement: Supplementary file 1 — Figure S1. Simulations. Estimated motion parameters from the synthesized k‐space with (A) and without (B) the addition of noise for the motion‐corrected and motion + B0‐corrected reconstruction. GT motion parameters are shown on the right. Labels for translation (Tr) are defined for the left–right (LR), anterior–posterior (AP) and foot‐head (FH) direction. Labels for rotation (Rot) are defined for rotation around the LR, AP and FH axis, respectively pitch, roll and yaw rotation Figure S2. Simulations in the absence of noise. Reconstruction performance regarding the LC maps droll (A) and dpitch (B). For each LC map, the GT used to generate simulated data (I) is compared to the estimated LC maps (II), together with corresponding residuals (III). Error maps are small inside the brain with localized errors outside (white arrow). For visual purposes, tissue is extracted by thresholding the corresponding magnitude images Figure S3. Simulations in the absence of noise: Reconstructed images (A) and the corresponding residuals with respect to the GT (B) for the uncorrected (I), motion‐corrected (II) and motion+B0‐corrected reconstruction (III) compared to the reference reconstructions (IV–V). Note that the displayed error range in (B) is 10% of the display range in (A) Figure S4. In‐vivo Experiment 2 (motion). Estimated LC maps droll (A) and dpitch (B) for subject 2 in the different controlled motion experiments (I–III). For visual purposes, tissue is extracted by thresholding the corresponding magnitude images Figure S5. In‐vivo Experiment 2 (motion). (I–III) Estimated motion traces for subject 2 in the different controlled motion experiments for the (A) motion‐corrected and (B) motion + B0‐corrected reconstruction. The horizontal time axis is removed from (A) to improve clarity. Labels for translation (Tr) are defined for the left–right (LR), anterior–posterior (AP) and foot‐head (FH) directions. Labels for rotation (Rot) are defined for rotation around the LR, AP and FH a [file MRM-88-817-s001.pdf]

Supporting Information 1: Taylor approximation of susceptibility-induced B0 fields

Susceptibility-induced B0 fields $B_0(\boldsymbol{r})$ (and hence the induced frequencies $\omega(\boldsymbol{r}) = \gamma B_0(\boldsymbol{r})$ where γ is the gyromagnetic ratio) originate from a shift-invariant convolution of the spatially varying susceptibility distribution $\chi(\boldsymbol{r})$ with the magnetic dipole kernel. Marques et al. (1) proposed a rapid calculation of $B_0(\boldsymbol{r})$ from $\chi(\boldsymbol{r})$ by expressing the convolution as a multiplication in the Fourier domain:

$$\omega(\boldsymbol{r}) = \gamma B_0(\boldsymbol{r}) \; = FT^{-1}\left(\gamma B_{0,\text{magnet}}\left(\frac{1}{3}-\frac{\boldsymbol{k}_z^2}{|\boldsymbol{k}|^2}\right)FT\big(\chi(\boldsymbol{r})\big)\right) \qquad [S1]$$

where $\boldsymbol{k}$ is the spectral coordinate, $B_{0,\text{magnet}}$ the main magnetic field and FT/FT^{-1} respectively the forward and inverse Fourier transform. Eq. S1 reveals the linear dependence of the susceptibility induced B0 on field strength ($B_{0,\text{magnet}}$). Pose-dependent B0 fields arising from a rigid transformation $T_{\boldsymbol{z}_n}$ of the head at segment n (defined by the transformation parameters $\boldsymbol{z}_n$consisting of 3 translations and 3 rotations $\boldsymbol{\theta}_n$) can be analysed by transforming the head susceptibility $\chi(\boldsymbol{r})$:

$$\omega_n(\boldsymbol{r},\boldsymbol{z}_n) = \gamma B_{0,n}(\boldsymbol{r},\boldsymbol{z}_n) = FT^{-1}\left(\gamma B_{0,\text{magnet}}\left(\frac{1}{3}-\frac{\boldsymbol{k}_z^2}{|\boldsymbol{k}|^2}\right)FT\left(T_{\boldsymbol{z}_n}\big(\chi(\boldsymbol{r})\big)\right)\right) \qquad [S2]$$

It should be noted that $T_{\boldsymbol{z}_n}$ is the analytical transformation corresponding to the transformation matrix $\boldsymbol{T}_{\boldsymbol{z}_n}$ used in the manuscript. It was shown that the pose-dependent B0 can be analysed in the object's co-ordinate frame by applying the transformation to the k-space coordinates instead of $\chi(\boldsymbol{r})$:

$$\omega_n(\boldsymbol{r},\boldsymbol{z}_n) = \gamma B0_n(\boldsymbol{r},\boldsymbol{z}_n) = FT^{-1}\left(\gamma B_{0,\text{magnet}}\left(\frac{1}{3}-\frac{\left[T_{\boldsymbol{\theta}_n}^{-1}(\boldsymbol{k})\right]_z^2}{\left|T_{\boldsymbol{\theta}_n}^{-1}(\boldsymbol{k})\right|^2}\right)FT\big(\chi(\boldsymbol{r})\big)\right) \qquad [S3]$$

where the inverse transformation is applied since only the relative rotation between $\chi(\boldsymbol{k})$ and $\boldsymbol{k}$ matters. Since shift-invariant convolutions are used, translations don't affect the B0 in the head co-ordinate frame and hence $T_{\boldsymbol{\theta}_n}^{-1}$ is used instead of $T_{\boldsymbol{z}_n}^{-1}$.

Applying the rotation to $\boldsymbol{k}$ has the advantage that $T_{\boldsymbol{\theta}_n}^{-1}$ $(\boldsymbol{k})$ has an analytical expression:

$$\begin{aligned} T_{\boldsymbol{\theta}_n}^{-1}(\boldsymbol{k}) &= \Big(\boldsymbol{R}_x(\theta_{n,\text{x}})\boldsymbol{R}_y(\theta_{n,\text{y}})\boldsymbol{R}_z(\theta_{n,\text{z}})\Big)^{-\mathbf{1}}\ \boldsymbol{k} \\ &= \begin{bmatrix} 1 & 0 & 0 \\ 0 & \cos(\theta_{n,\text{x}}) & -\sin(\theta_{n,\text{x}}) \\ 0 & \sin(\theta_{n,\text{x}})\ 0 & \cos(\theta_{n,\text{x}}) \end{bmatrix} \end{aligned} \qquad [S4]$$

$$* \begin{bmatrix} \cos(\theta_{n,\mathrm{y}}) & 0 & \sin(\theta_{n,\mathrm{y}}) \\ 0 & 1 & 0 \\ -\sin(\theta_{n,\mathrm{y}}) & 0 & \cos(\theta_{n,\mathrm{y}}) \end{bmatrix}$$

$$* \begin{bmatrix} cos(\theta_{n,\mathrm{z}}) & -sin(\theta_{n,\mathrm{z}}) & 0 \\ sin(\theta_{n,\mathrm{z}}) & cos(\theta_{n,\mathrm{z}}) & 0 \\ 0 & 0 & 1 \end{bmatrix} \begin{bmatrix} k_x \\ k_y \\ k_z \end{bmatrix}$$

Extracting the z-component of $T_{\boldsymbol{\theta}_n}^{-1}(\boldsymbol{k})$ and raising it to the power of 2 gives:

$$\left[T_{\boldsymbol{\theta}_n}^{-1}(\boldsymbol{k})\right]_z^2 = \left[k_x \sin(\theta_{n,\mathrm{y}}) + k_z \cos(\theta_{n,\mathrm{x}}) \cos(\theta_{n,\mathrm{y}}) - k_y \cos(\theta_{n,\mathrm{y}}) \sin(\theta_{n,\mathrm{x}})\right]^2 \qquad \text{[S5]}$$

Inserting Eq. S5 into Eq. S3 and noting that $\left|T_{\boldsymbol{\theta}_n}^{-1}(\boldsymbol{k})\right|^2 = |\boldsymbol{k}|^2$ obtains the following expression:

$$\omega_n(\boldsymbol{r}, \boldsymbol{\theta}_n) = FT^{-1}\left(\gamma B_{0,\mathrm{magnet}} \left(\frac{1}{3} - \frac{\left[k_x \sin(\theta_{n,\mathrm{y}}) + k_z \cos(\theta_{n,\mathrm{x}}) \cos(\theta_{n,\mathrm{y}}) - k_y \cos(\theta_{n,\mathrm{y}}) \sin(\theta_{n,\mathrm{x}})\right]^2}{|\boldsymbol{k}|^2}\right) FT(\chi(\boldsymbol{r}))\right) \qquad \text{[S6]}$$

Eq. S6 allows $\omega_n(\boldsymbol{r}, \boldsymbol{z}_n)$ to be estimated implicitly via estimating $\chi(\boldsymbol{r})$. Although this is interesting from a data-efficiency approach (only estimating $\chi(\boldsymbol{r})$), this problem is extremely ill-conditioned. Therefore, as previously proposed (1), we resort to a 1st order Taylor expansion of Eq. S6 with respect to rotation parameters $\boldsymbol{\theta}_n$:

$$\begin{aligned} \omega_n(\boldsymbol{r}, \boldsymbol{\theta}_n) &\cong \omega_n(\boldsymbol{r}, \boldsymbol{\theta}_n = \boldsymbol{0}) + \frac{\partial \omega_n(\boldsymbol{r}, \boldsymbol{\theta}_n)}{\partial \boldsymbol{\theta}_n} \boldsymbol{\theta}_n \\ &= FT^{-1}\left(\gamma B_{0,\mathrm{magnet}} \left(\frac{1}{3} - \frac{\boldsymbol{k}_z^2}{|\boldsymbol{k}|^2}\right) FT(\chi(\boldsymbol{r}))\right) \\ &\quad + FT^{-1}\left(\gamma B_{0,\mathrm{magnet}} \left(\frac{-2k_y k_z}{|\boldsymbol{k}|^2}\right) FT(\chi(\boldsymbol{r}))\right) \theta_{n,\mathrm{x}} \\ &\quad + FT^{-1}\left(\gamma B_{0,\mathrm{magnet}} \left(\frac{+2k_x k_z}{|\boldsymbol{k}|^2}\right) FT(\chi(\boldsymbol{r}))\right) \theta_{n,\mathrm{y}} \end{aligned} \qquad \text{[S7]}$$

Which can be re-written more compactly as:

$$\omega_n(\boldsymbol{r},\boldsymbol{\theta}_n) \;=\; \omega_n(\boldsymbol{r},\boldsymbol{\theta}_n = \boldsymbol{0}) \;+\; d_{\mathrm{x}}(\boldsymbol{r})\theta_{n,\mathrm{x}} + d_{\mathrm{y}}(\boldsymbol{r})\theta_{n,\mathrm{y}} \qquad \text{[S8]}$$

where $d_{\mathrm{x}}(\boldsymbol{r})$ and $d_{\mathrm{y}}(\boldsymbol{r})$ are the linear coefficient (LC) maps in respectively x and y rotation angles $\theta_{n,\mathrm{x}}$ and $\theta_{n,\mathrm{y}}$:

$$\begin{aligned} d_{\mathrm{x}}(\boldsymbol{r}) &\triangleq FT^{-1}\left(\gamma B_{0,\mathrm{magnet}}\left(\frac{-2k_y k_z}{|\boldsymbol{k}|^2}\right)FT\big(\chi(\boldsymbol{r})\big)\right) \\ d_{\mathrm{y}}(\boldsymbol{r}) &\triangleq FT^{-1}\left(\gamma B_{0,\mathrm{magnet}}\left(\frac{+2k_x k_z}{|\boldsymbol{k}|^2}\right)FT\big(\chi(\boldsymbol{r})\big)\right) \end{aligned} \qquad \text{[S9]}$$

Note that the z rotation angle $\theta_{n,\mathrm{z}}$ does not appear in Eq. S8 since the magnetic dipole kernel is symmetric around the z axis (by convention the direction of the main magnetic field). As mentioned already, translations don't contribute either since the model in Eq. S1 is based on a shift-invariant convolution.

Choosing the x and y axis respectively the left-right (LR) axis and posterior-anterior (PA) axis results in the terminology adapted in the manuscript:

$$\omega_n(\boldsymbol{r},\boldsymbol{\theta}_n) \;=\; \omega_n(\boldsymbol{r},\boldsymbol{\theta}_n = \boldsymbol{0}) \;+\; d_{\mathrm{pitch}}(\boldsymbol{r})\theta_{n,\mathrm{pitch}} + d_{\mathrm{roll}}(\boldsymbol{r})\theta_{n,\mathrm{roll}} \qquad \text{[S10]}$$

As pointed out in the manuscript, the 0th order term $\omega_n(\boldsymbol{r},\boldsymbol{\theta}_n = \boldsymbol{0})$ is pose-independent and can therefore be embedded in the complex image $\boldsymbol{x}$ in Eq. 2 when using SPGR sequences (2). Removing the 0th order term from Eq. S10 yields:

$$\omega_n(\boldsymbol{r},\boldsymbol{\theta}_n) \;=\; d_{\mathrm{pitch}}(\boldsymbol{r})\theta_{n,\mathrm{pitch}} + d_{\mathrm{roll}}(\boldsymbol{r})\theta_{n,\mathrm{roll}} \qquad \text{[S11]}$$

Since the dependence on the rotation angles $\boldsymbol{\theta}_n$ is implicitly contained in the subscript n, it will be left out for simplicity: $\omega_n(\boldsymbol{r},\boldsymbol{\theta}_n) \rightarrow \omega_n(\boldsymbol{r})$:

$$\omega_n(\boldsymbol{r}) \;=\; d_{\mathrm{pitch}}(\boldsymbol{r})\theta_{n,\mathrm{pitch}} + d_{\mathrm{roll}}(\boldsymbol{r})\theta_{n,\mathrm{roll}} \qquad \text{[S12]}$$

Eq. S12 is now identical to Eq. 4 in the manuscript.

Supporting Information 2: Algorithm implementation details

A MATLAB implementation to reproduce the experiments is made available at https://github.com/mriphysics/B0InformedDISORDER. The proposed method is implemented building upon the repository used in DISORDER (3) (https://github.com/mriphysics/DISORDER). Modifications of the algorithm with respect to the latter are listed below:

Added features:

- Signal model: The signal model is extended with the pose-dependent B_0 fields as described in Eq. 4 and Eq. 5 in the manuscript. Since rotation angles in Eq. 4 are defined with respect to the scanner co-ordinates (namely pitch and roll), rotation angles used for motion correction (in logical coordinates) are converted to world co-ordinates when used in the B0 model.
- GD optimisation of LC maps: A line search strategy is adopted for Gradient Descent (GD) updates to ensure a reduced data-consistency loss. The GD optimisation is aborted when no appropriate step size is found or when the data-consistency loss goes below a given threshold (10^{-5} in this work). Since the gradient formulation Eq. A5 contains a multiplication with $\boldsymbol{D}(\boldsymbol{x}^{\boldsymbol{H}})$, voxels with stronger signal are favoured. This can result in poor LC estimates in areas with low signal. Therefore, the gradient used in our GD optimisation does not include this $\boldsymbol{D}(\boldsymbol{x}^{\boldsymbol{H}})$ multiplication.
- Regularisation:
  - Based on observations in the motion-free dataset (Figure 1), smoothness can be enforced for $\boldsymbol{d}_{\text{pitch}}$ and $\boldsymbol{d}_{\text{roll}}$ and is implemented by filtering the gradient for $\boldsymbol{d}$ with a low-pass filter of 1.5 mm. This implementation was preferred over the more common finite differences regularisation (4) since the latter requires dynamic hyper-parameter tuning between resolution levels and array sizes.
  - In areas with very strong LC values (in Hz/degree), the B0 induced phase from non-zero rotation angles might exhibit phase wraps in areas close to air-tissue interfaces. This can affect the LC values in those voxels and make them inconsistent with surrounding voxels (which normally have lower LC values and less prone to this issue). A phase unwrapping strategy is adopted to correct these incorrectly estimated LC values and make them consistent with surrounding voxels: for an estimate of LC maps, B0 induced phase is

generated for a range of rotation angles (-15 to 15 degrees). Next, each individual phase map is unwrapped. Afterwards, LC maps are estimated from these unwrapped phase maps for each voxel independently. This approach should only change estimated LC values with strong inconsistencies with surrounding voxels (non-smooth LC maps). This regularisation is performed every 5 outer iterations.

Modified features:

- Alternating optimisation scheme: For every outer iteration in Eq. 6 in the manuscript, the image $\boldsymbol{x}$ and motion parameters $\boldsymbol{z}_n$ are updated using one iteration whereas $\boldsymbol{d}$ is updated twice as the GD algorithm has slower convergence properties compared to respectively the Conjugate Gradient (CG) and Levenberg-Marquardt (LM) algorithms. The alternating optimisation is aborted when the LM algorithm has reached convergence for every segment of k-space within a single iteration. The final CG image reconstruction is performed using 300 iterations.
- Multi-resolution approach: The maximum resolution used for initial motion estimation is 6 mm (4 mm in (3)) since the B_0-induced non-convexness of the forward model can make the optimisation more prone to local minima at increased resolution. It was also observed that early GD updates without correct motion parameters obtained unrealistic $\boldsymbol{d}$ estimates, supported by the linear dependence of the GD update on pitch and roll rotation angles (Eq. A6 in the manuscript). Therefore, the estimation of $\boldsymbol{d}$ is only activated after 6 outer iterations of the first resolution level, after which the motion estimates are more reliable.

Disabled features:

- Motion compression: Motion binning was disabled.
- Regularisation: Regularisation in the image reconstruction was disabled to isolate the performance of our proposed signal model from the effect of advanced image regularisation techniques.

Supporting Information 3: Simulations

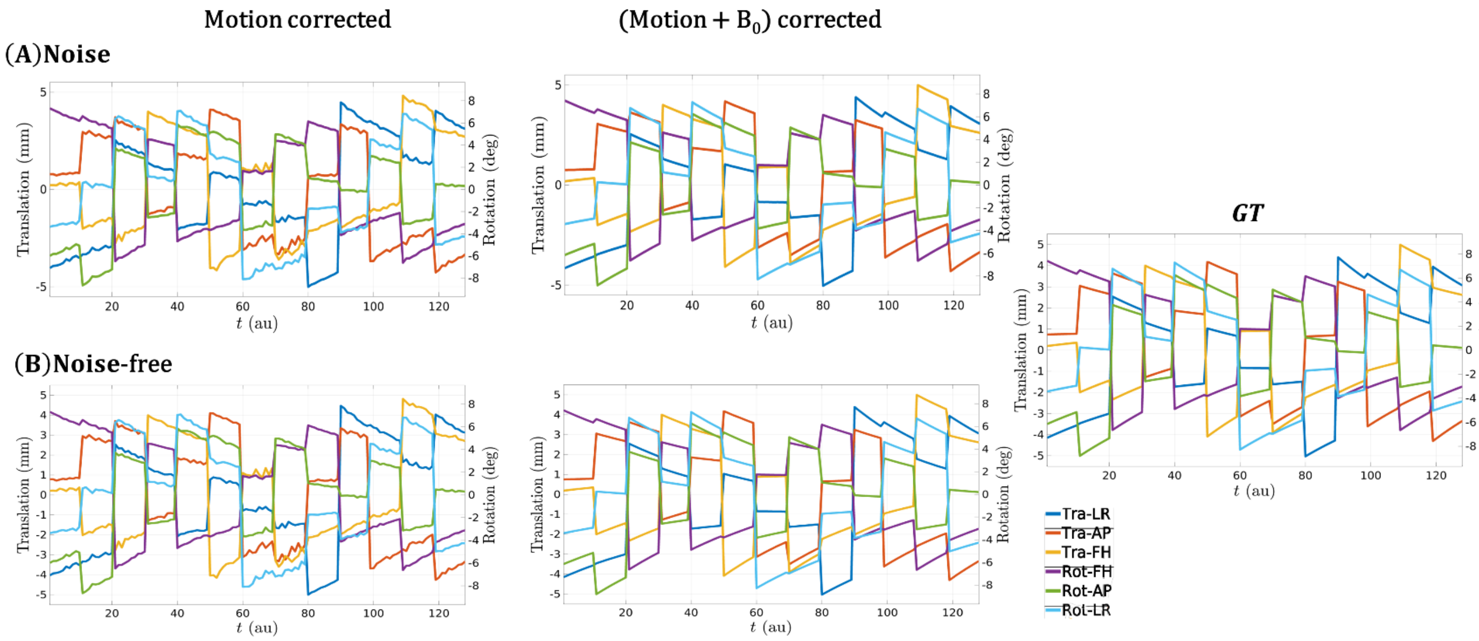

Figure S1: *Simulations*: Estimated motion parameters from the synthesized k-space with (A) and without (B) the addition of noise for the motion-corrected and (motion + B_0) corrected reconstruction. GT motion parameters are shown on the right. Labels for translation (Tr) are defined for the left-right (LR), anterior-posterior (AP) and foot-head (FH) direction. Labels for rotation (Rot) are defined for rotation around the LR, AP and FH axis, respectively pitch, roll and yaw rotation. Improvements in the motion parameters are indicated by the red markers.

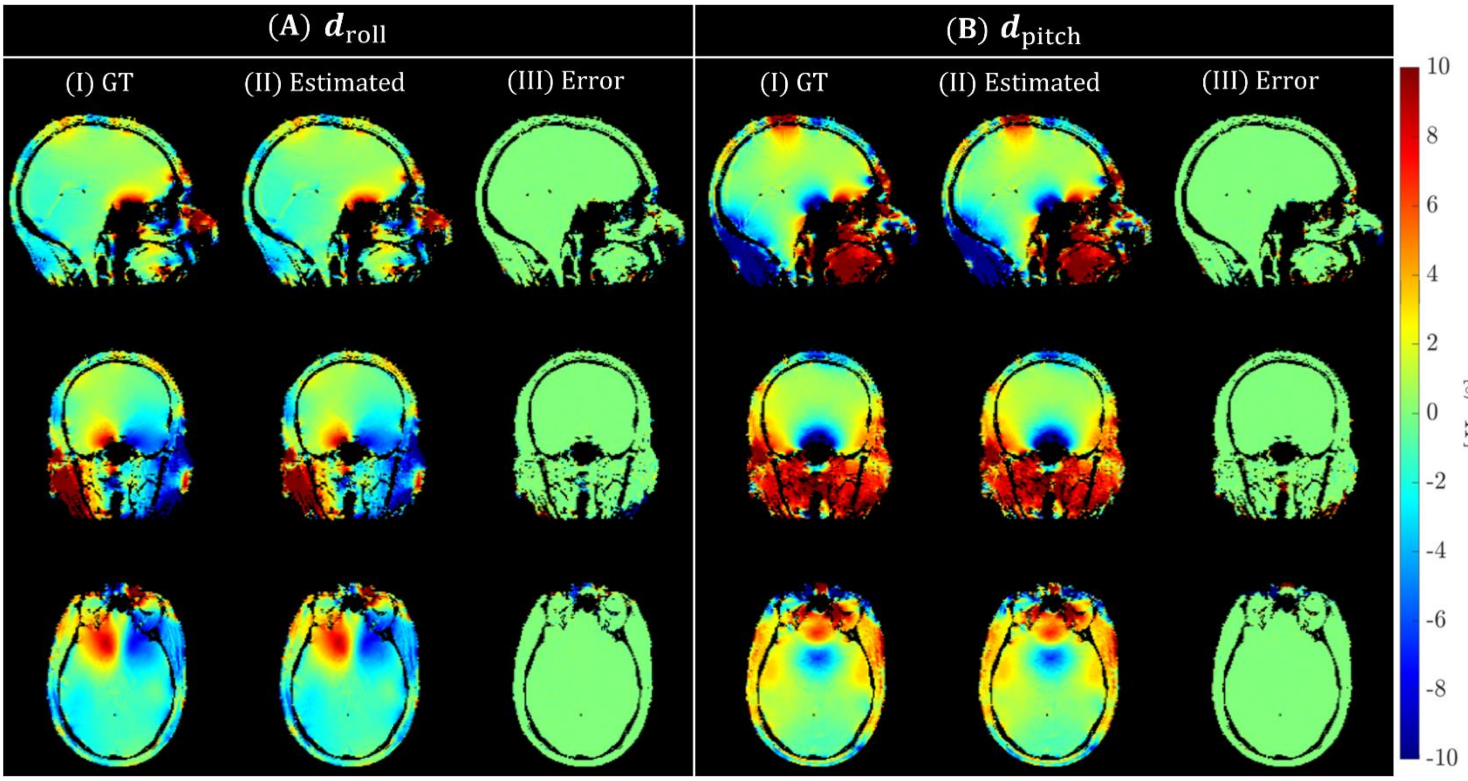

Figure S2: *Simulations in the absence of noise*: Reconstruction performance regarding the LC maps d_{roll} (A) and d_{pitch} (B). For each LC map, the GT used to generate simulated data (I) is compared to the estimated LC maps (II), together with corresponding residuals (III). Error maps are small inside the brain with localised errors outside. For visual purposes, tissue is extracted by thresholding the corresponding magnitude images.

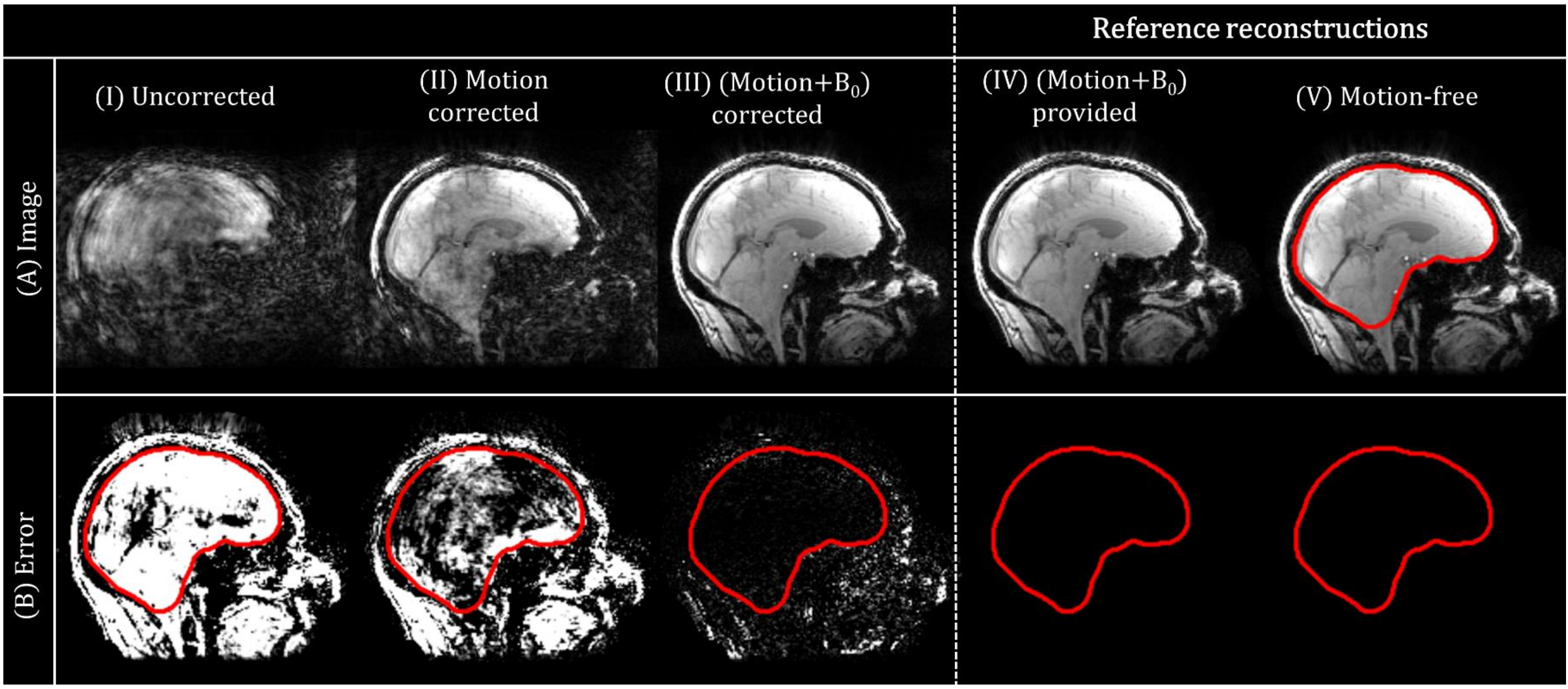

Figure S3: *Simulations in the absence of noise*: Reconstructed images (A) and the corresponding residuals with respect to the GT (B) for the uncorrected (I), motion corrected (II) and (motion+B_0) corrected reconstruction (III) compared to the reference reconstructions (IV-V). Note that the displayed error range in (B) is 10% of the display range in (A).

Supporting Information 4: In vivo reconstructions

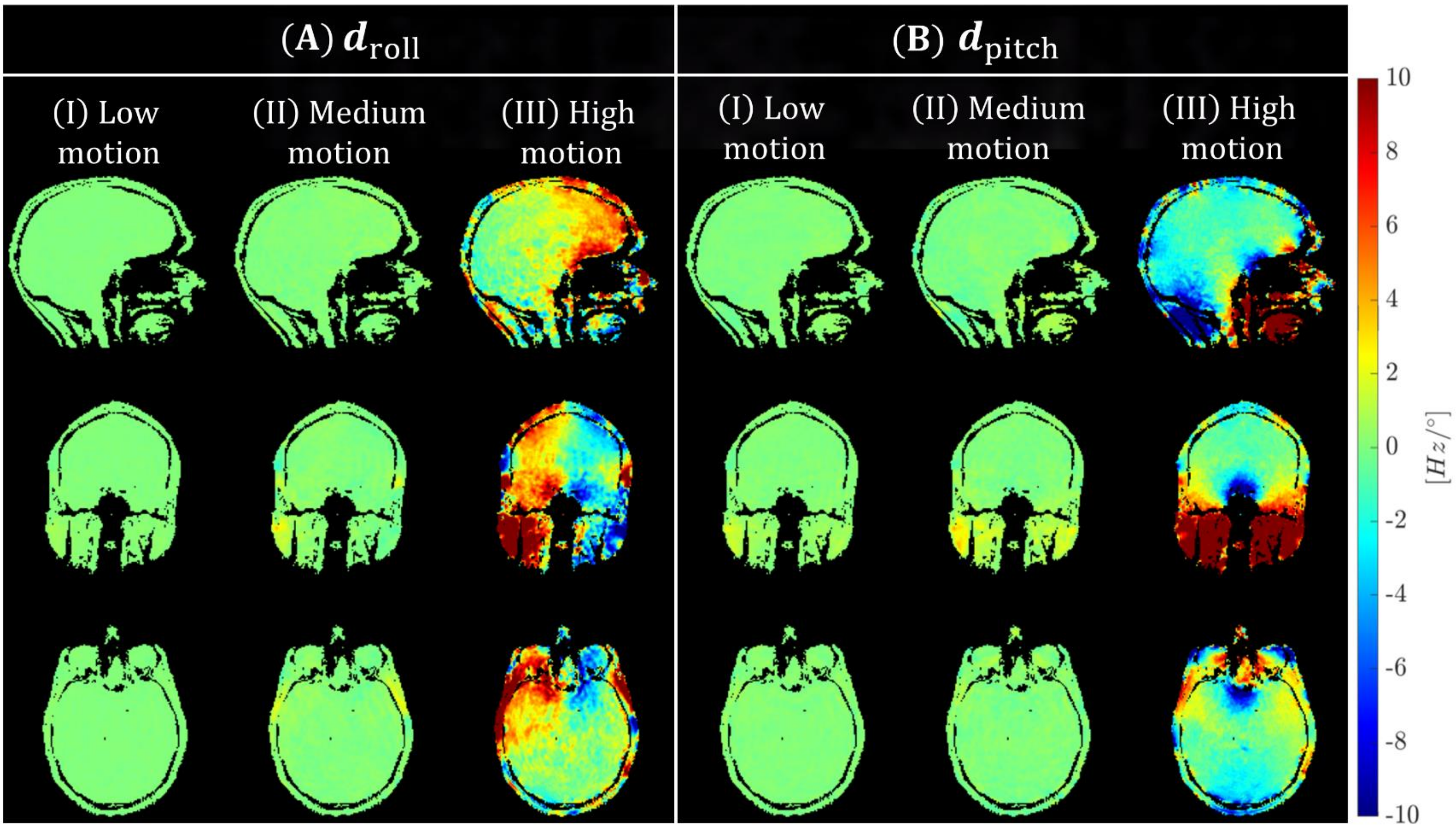

Figure S4: *In-vivo Experiment 2 (motion):* Estimated LC maps $\boldsymbol{d_{roll}}$ (A) and $\boldsymbol{d_{pitch}}$ (B) for subject 2 in the different controlled motion experiments (I-III). For visual purposes, tissue is extracted by thresholding the corresponding magnitude images.

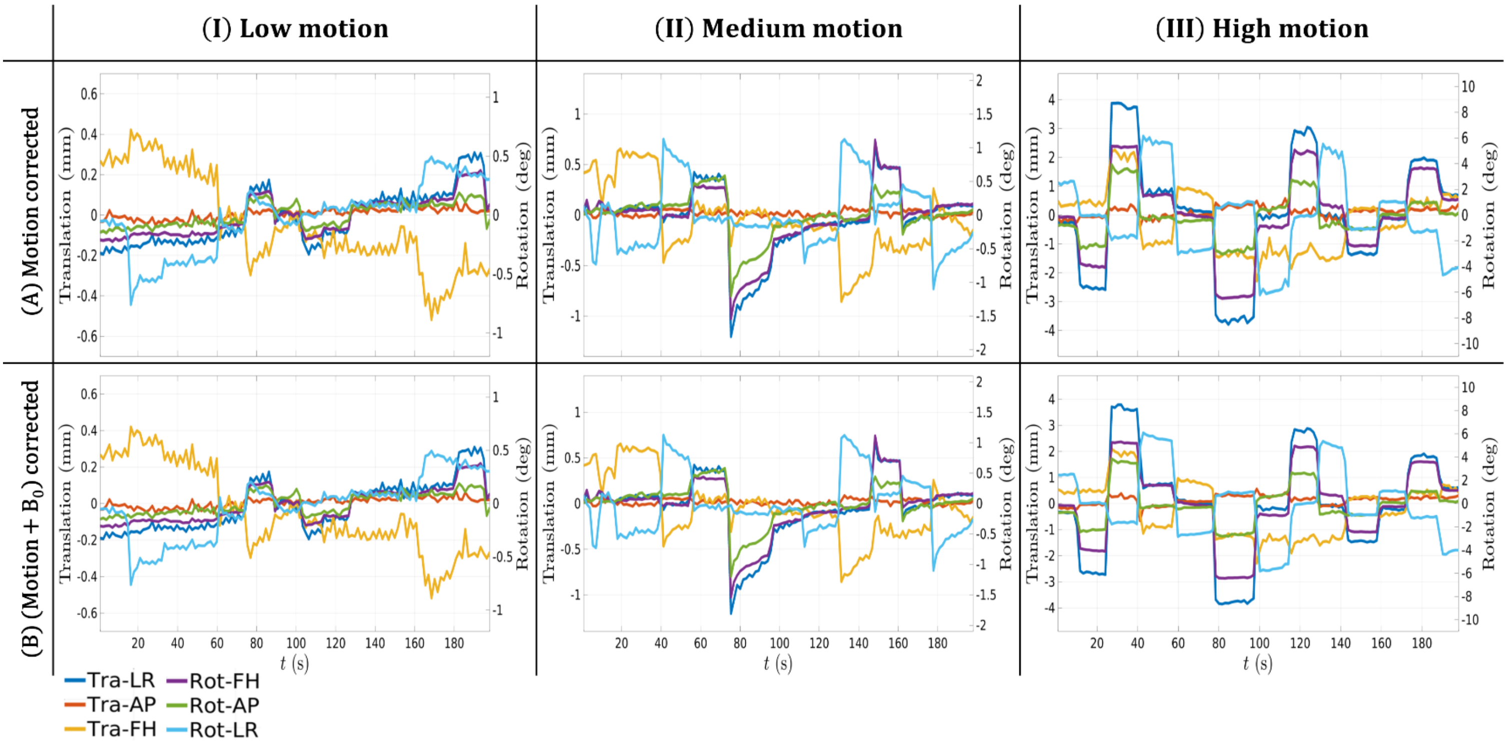

Figure S5: *In-vivo Experiment 2 (motion):* (I-III) Estimated motion traces for subject 2 in the different controlled motion experiments for the (A) motion-corrected and (B) motion + B0 corrected reconstruction. The horizontal time axis is removed from (A) to improve clarity. Labels for translation (Tr) are defined for the left-right (LR), anterior-posterior (AP) and foot-head (FH) directions. Labels for rotation (Rot) are defined for rotation around the LR, AP and FH axis, respectively pitch, roll and yaw rotation.

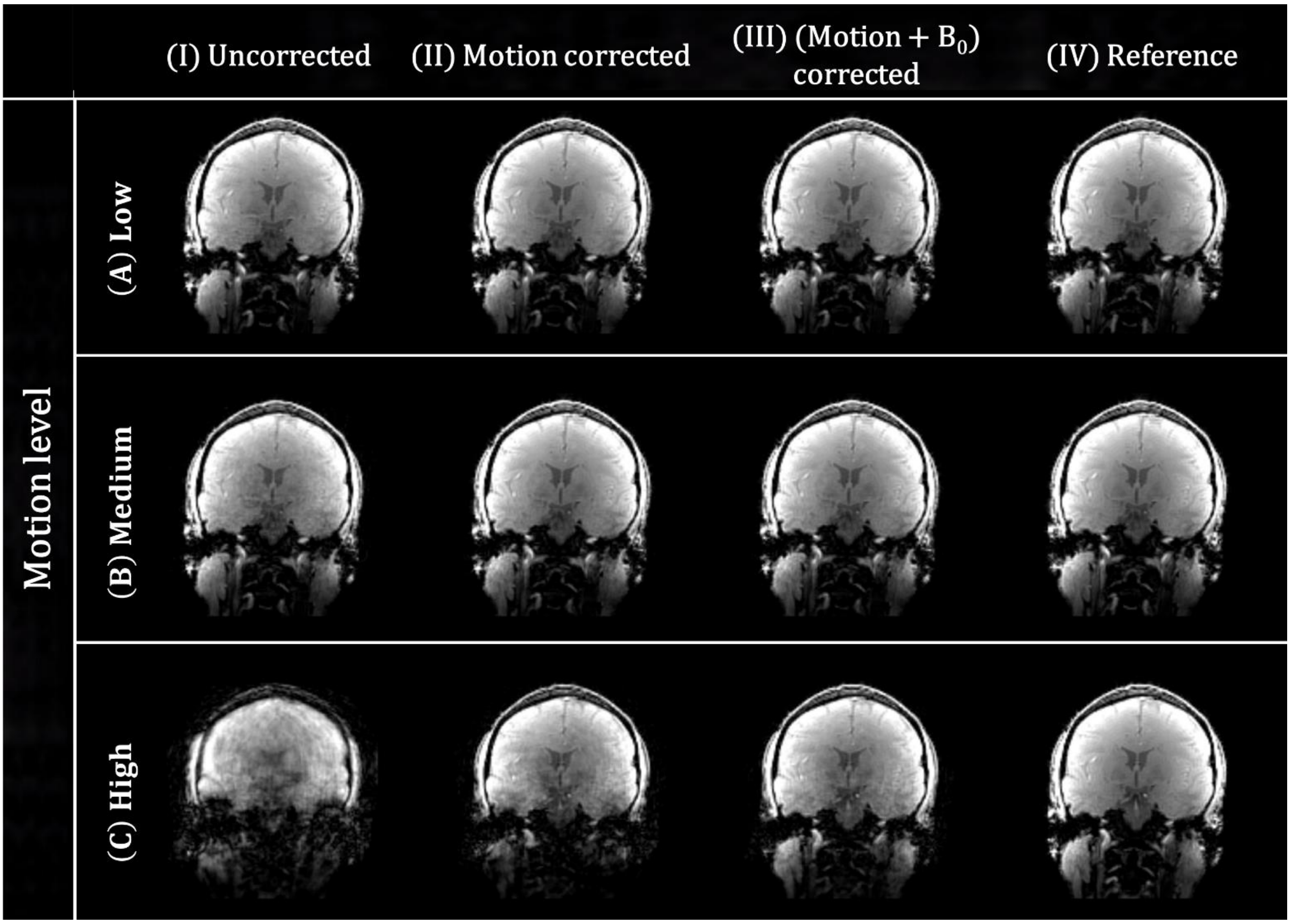

Figure S6: *In-vivo Experiment 2 (motion):* Coronal view of the reconstructed images for subject 2. The different sub-experiments are shown in the rows (A-C) and the different reconstruction methods are shown in the columns (I-III). A motion free scan from Experiment 1 is added as a reference (IV).

Supporting Information References

1. Marques JP, Bowtell R. Application of a fourier-based method for rapid calculation of field inhomogeneity due to spatial variation of magnetic susceptibility. Concepts in Magnetic Resonance Part B: Magnetic Resonance Engineering. 2005;25(1):65–78.

2. Elster AD. Gradient-echo MR imaging: Techniques and acronyms. Radiology. 1993;186(1):1–8.

3. Cordero-Grande L, Ferrazzi G, Teixeira RPAG, O'Muircheartaigh J, Price AN, Hajnal J v. Motion-corrected MRI with DISORDER: Distributed and incoherent sample orders for reconstruction deblurring using encoding redundancy. Magnetic Resonance in Medicine. 2020;84(2):713–26.

4. Sutton BP, Noll DC, Fessler JA. Dynamic field map estimation using a spiral-in/spiral-out acquisition. Magnetic Resonance in Medicine. 2004;51(6):1194–204.
